# Numerical ragweed pollen forecasts using different source maps: a comparison for France

**DOI:** 10.1007/s00484-016-1188-x

**Published:** 2016-06-18

**Authors:** Katrin Zink, Pirmin Kaufmann, Blaise Petitpierre, Olivier Broennimann, Antoine Guisan, Eros Gentilini, Mathias W. Rotach

**Affiliations:** 1 Institute of Meteorology and Geophysics, University of Innsbruck, 6020 Innsbruck, Austria; 2 Federal Office of Meteorology and Climatology MeteoSwiss, 8044 Zürich, Switzerland; 3 Department of Ecology, Evolution, University of Lausanne, 1015 Lausanne, Switzerland; 4 Institute of Earth Surface Dynamics, University of Lausanne, 1015 Lausanne, Switzerland

**Keywords:** Ragweed, Distribution map, Land use, Ragweed inventory, Pollen, Numerical simulation

## Abstract

One of the key input parameters for numerical pollen forecasts is the distribution of pollen sources. Generally, three different methodologies exist to assemble such distribution maps: (1) plant inventories, (2) land use data in combination with annual pollen counts, and (3) ecological modeling. We have used six exemplary maps for all of these methodologies to study their applicability and usefulness in numerical pollen forecasts. The ragweed pollen season of 2012 in France has been simulated with the numerical weather prediction model COSMO-ART using each of the distribution maps in turn. The simulated pollen concentrations were statistically compared to measured values to derive a ranking of the maps with respect to their performance. Overall, approach (2) resulted in the best correspondence between observed and simulated pollen concentrations for the year 2012. It is shown that maps resulting from ecological modeling that does not include a sophisticated estimation of the plant density have a very low predictive skill. For inventory maps and the maps based on land use data and pollen counts, the results depend very much on the observational site. The use of pollen counts to calibrate the map enhances the performance of the model considerably.

**Electronic Supplementary Material:**

The online version of this article (doi:10.1007/s00484-016-1188-x) contains supplementary material, which is available to authorized users.

## Introduction

Airborne pollen grains can lead to allergenic reactions (such as rhinitis or asthma) in sensitized persons. Even though medication is possible, avoidance of the allergens is the best way to avoid symptoms (van Moerbeke 1997). Therefore, spatially and temporally highly resolved pollen forecasts are needed to help allergy sufferers to plan their outdoor activities. Traditional pollen forecasts are issued manually, taking into account measured pollen concentrations of the previous days, climatological knowledge about the typical course of the pollen season, and current weather forecasts. In recent years, numerical weather prediction (NWP) models have been extended to simulate the dispersion of pollen grains (e.g., Vogel et al. 2008; Sofiev et al. 2006; Efstathiou et al. 2011; Zhang et al. 2014). One of the major sources of uncertainty in these models is the distribution of pollen sources (Sofiev et al. 2006; Skjøth et al. 2010). Therefore, the mapping of plant distributions needs to be done more accurately.

Different ways to map the distribution of pollen sources have been described in the literature. The first methodology relies on inventory data displaying the occurrence of the allergenic plant. This has been applied to trees (e.g., Sofiev et al. 2006; Skjøth et al. 2008) and ragweed (Bullock et al. 2012; Zink et al. 2012). The main obstacle of this approach is the limited availability, completeness, comparability, and timeliness of such data. For example, forest inventories only cover forested areas even though trees can also grow outside of forests. Additionally, the quantitative information of inventories varies strongly regarding the degree of detail (e.g., classified information or exact number of plants) and the accuracy due to the varying methods of estimation.

A second methodology combines land use data and observed annual pollen counts to derive a plant distribution (e.g., Skjøth et al. 2010; Thibaudon et al. 2014). In addition to land use and pollen counts, Pauling et al. (2012) take into account forest inventory data. This methodology assumes that the same land use class is equally suitable for the plant in question throughout the entire model domain. With respect to NWP modeling when used for numerical pollen forecasts, this translates to extended regions as large as ’whole Europe’ with quite substantially different climatic zones and hence growing conditions for plant species. The limitations of this method are the availability of pollen measurements, the spatial resolution, timeliness, and the detail of the classification of the land use data set. Furthermore, when using annual pollen counts for the local calibration of the map, this method cannot be applied to regions whose annual pollen counts are dominated by transported pollen grains.

A third methodology is based on ecological modeling: taking into account biological, climatological, geographical, and anthropogenic factors, the spread of the plant is simulated (e.g., Bullock et al. 2012; Prank et al. 2013). In Prank et al. (2013), the simulated plant distribution is calibrated using a numerical dispersion model: the pollen concentrations are modeled using the original map as input for the dispersion model. The simulated pollen concentrations are then compared to those observed and the deviations are used to tune the map. This calibration procedure is repeated until the differences between the simulated and observed concentrations fall below a certain threshold. The limitations of the ecological modeling are related to the underlying assumptions and availability, completeness and quality of data that is used during the building-process of the model (e.g., plant inventories). Additionally, anthropogenic influences can only be taken into account in a stochastic manner, so that the resulting map is always only one out of many possible solutions. The calibration process using a dispersion model relates all the differences between measured and simulated pollen concentrations to the source map, neglecting the uncertainties coming from the parameterization of pollen emission (Zink et al. 2013), or the simulation of transport processes. Additionally, it assumes that both measured and simulated pollen concentrations are mainly influenced by local pollen emission. Hence, this procedure is of limited use in areas that are mainly influenced by transported pollen.

As all of these methods have their specific advantages and disadvantages, the question arises which method is best for generating distribution maps used for numerical pollen forecasts. We address this question by simulating ragweed (*Ambrosia artemisiifolia* L.) pollen concentrations using maps that represent all three methodologies in turn. We have chosen France as the region of study since ragweed distribution maps of all three types were available for this area. A total of six different distribution maps has been used to run the NWP system COSMO-ART (**Co**nsortium for **S**mall-scale **Mo**delling-**A**erosols and **R**eactive **T**race Gases, details can be found in the Chapter “Simulations using the model COSMO-ART”) and simulate ragweed pollen concentrations for the pollen season of 2012. These are then statistically compared to observed pollen concentrations. The results are used to assess the suitability of the different maps in numerical pollen forecasts. A ranking of the maps is derived according to the performance of the different forecasts. Finally, a recommendation is given on how to generate a distribution map for numerical pollen forecasts.

Additional material, including a list of acronyms, can be found in the supplement.

## Materials and methods

### Two distribution maps based on inventory data

**Fig. 1:**
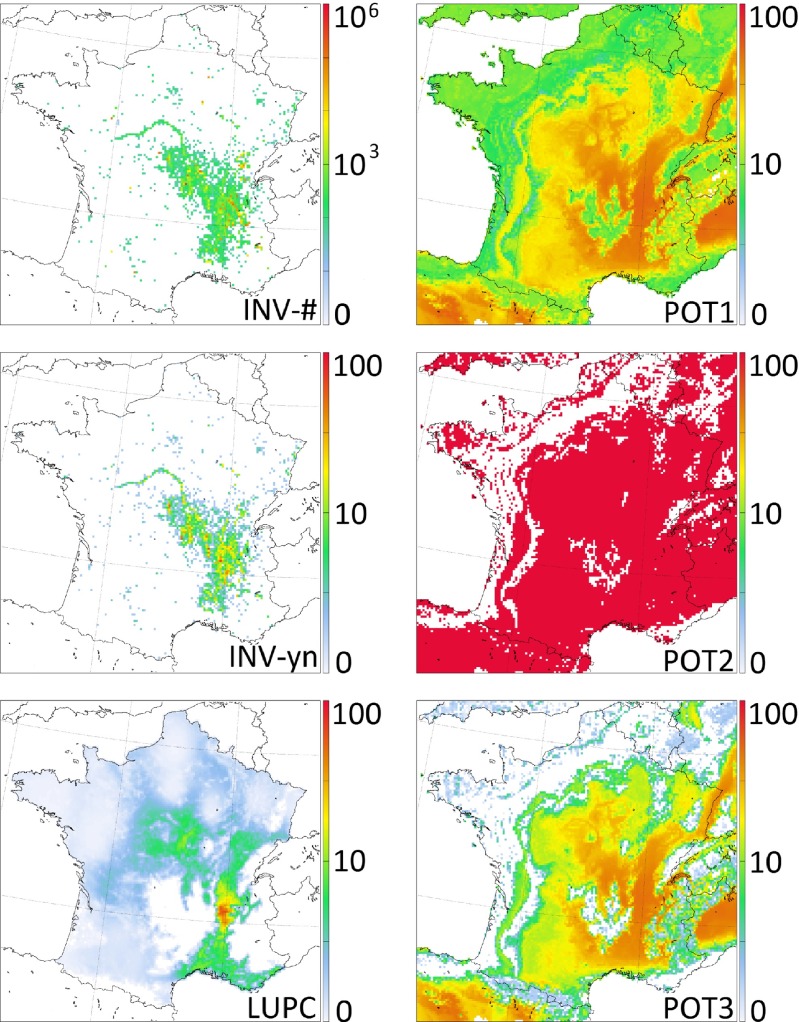
The six different ragweed distributions for France. The colors denote the quantity: for map INV-# the number of plants per grid cell, for map INV-yn the numbers of populations per grid cell, for map LUPC the plant density in %, for the potential maps (POT1, POT2, and POT3) the suitability of the grid cell transformed into a plant density in %. The images show the original maps before they were calibrated using part of the NWP simulations

We have used a ragweed distribution data set from France to create a source map for pollen emission. The data has been collected by the Fédération des conservatoires botaniques nationaux (FCBN) and has been published in Petermann (2011). The data gives the location and classified quantitative information for each plant stand. The inventory data was used to generate two distribution maps (see upper two images in the left column of Fig. 1):

- The full information given in the inventory was used to generate map ’INV-#’ (**inv**entory, numbered (**#**) quantitative information). This distribution map was created by counting all plants recorded within each grid cell of the model domain.
- According to Zink (2014), the quantitative information given in the plant inventories does not (generally) promote the skill of numerical pollen forecasts. Because of this, we disregarded the quantitative information given in the inventory to generate a second distribution map ’INV-yn’ (**inv**entory, **y**es-**n**o information). Here, the map to be used in the NWP model is generated by counting the number of plant localities within each grid cell of the model domain.

### A distribution map based on land use data

Thibaudon et al. (2014) have published a ragweed distribution map for France based on land use data and observations of airborne pollen concentrations. The method involves the following steps:

1. Based on ecological knowledge, the different classes of a land use data set are divided into two categories: suitable and unsuitable for ragweed growth.
2. For each grid cell of the distribution map, the percentage of the area suitable for ragweed growth is calculated based on the land use data set (which has a finer resolution than the distribution map).
3. For each observational site of pollen concentrations in the study area, the local ragweed density is calculated taking into account the percentage of suitable land use classes within a radius of 30 km and the mean Seasonal Pollen Index of the site (SPI, i.e., the total number of pollen measured during 1 year).
4. These local ragweed densities are interpolated onto the suitable land use areas using inverse distance weighting.
5. Grid cells above a certain altitude are set to zero plants, assuming that ragweed populations cannot reproduce at higher altitudes.

This map is called ’LUPC’ (**l**and **u**se and **p**ollen **c**ounts) in the following (see lower image in the left column of Fig. 1).

### Three potential distribution maps

Species distribution models (SDMs, Guisan and Thuiller 2005, more information is available in the supplementary materials) were calibrated using occurrences of ragweed from various herbarium and environmental agencies in France and neighboring countries (e.g., Switzerland, Germany, Austria, Slovenia, and Croatia) where ragweed can accomplish a full reproduction cycle (Storkey et al. 2014). Within the boundaries of these countries, 10,000 points were randomly sampled to depict the climatological conditions of the regions where ragweed is not present. The occurrences (and the randomly sampled non-occurrences) were related to six climate variables known to be important for the delineation of the potential distribution of ragweed: maximum temperature of the warmest month, annual temperature range, mean temperature of the coldest season, precipitation of the driest season, precipitation of the warmest season and aridity. Different modeling techniques were used: generalized linear models (GLM), generalized boosted regression models (GBM), and the maximum entropy method (Maxent). The predictions of these models were averaged following an ensemble approach (Thuiller et al. 2009). Modeling was achieved using the R package ’biomod2’ (Thuiller et al. 2009), keeping the default set of parameters. Models were calibrated on 70 % and evaluated with the remaining 30 % of the data. The modeling procedure was replicated 10 times and the final results consist in an average of the 10 replicates. We further performed a Multivariate Environmental Similarity Surfaces analysis (MESS, Elith et al. 2010) to identify climates ’non-analog’ to the calibration dataset. In this context, ’non-analog’ means that the specific climate might be suitable for ragweed, but it does not exist in the calibration data set. Finally, we derived three potential distribution maps from the raw predictions of the ensemble model (see right column of Fig. 1):

- POT1: continuous predictions of the ensemble model over the study area, with grid cells where at least one variable is non-analog according to the MESS analysis and grid cells without predictions (i.e., over water bodies) set to zero. The raw output of the model gives the suitability of the grid cell for ragweed growth on a scale between 0 and 1000. With the assumption that the actual plant density is linearly correlated with the suitability, the output of the model was then transformed into densities by rescaling the values between 0 % and 100 %. This leads to a maximum density of 55.3 % in the model domain.
- POT2: binary predictions derived from POT1 using a threshold set to ensure that 95 % of presences are predicted correctly. The assumption here is that below this threshold ragweed cannot grow and the density of ragweed at the corresponding grid cells is 0 %. At the grid cells suitable for ragweed, the density is set to 100 %.
- POT3: same as POT1 but with grid cells set to zero where POT2 is zero. In order to avoid strong gradients of the ragweed density at the borders of the regions that are set to zero, the remaining non-zero values of POT3 were linearly rescaled such that the maximum possible value (suitability of 1000) is set to 100 % and the lowest occurring value in POT3 (suitability of 82) is set to 0 %. This procedure leads to a maximum density of 51.3 % in the model domain.

### Simulations using the model COSMO-ART

The purpose of generating these distribution maps is to use them in numerical pollen forecasts. Therefore, in addition to comparing the maps themselves, we used the maps as input fields in the emission parameterization of the NWP system COSMO-ART to simulate the ragweed pollen season of 2012 in France. COSMO is a NWP model that has been initially developed at the German weather service DWD (Steppeler et al. 2002)). The extension ART (**A**erosols and **R**eactive **T**race Gases, Vogel et al. (2009)) is coupled to COSMO in order to allow the simulation of airborne substances/particles and their feedback on the weather system. The emission of pollen grains is parameterized according to Zink et al. (2013). The setup of the simulations is taken from Zink (2014).

The simulation of pollen dispersion using an online-coupled meteorology and transport model is computationally demanding. Even on a massively parallel high performance computing system, it took about 20 min laps time for 1 day simulated time. Thus, simulating the pollen season of 2012 took nearly 4 months. Unfortunately, it was not possible to use more computer resources for this project. Therefore, we were only able to simulate one season of pollen concentrations.

### Observational data of pollen concentrations

**Fig. 2:**
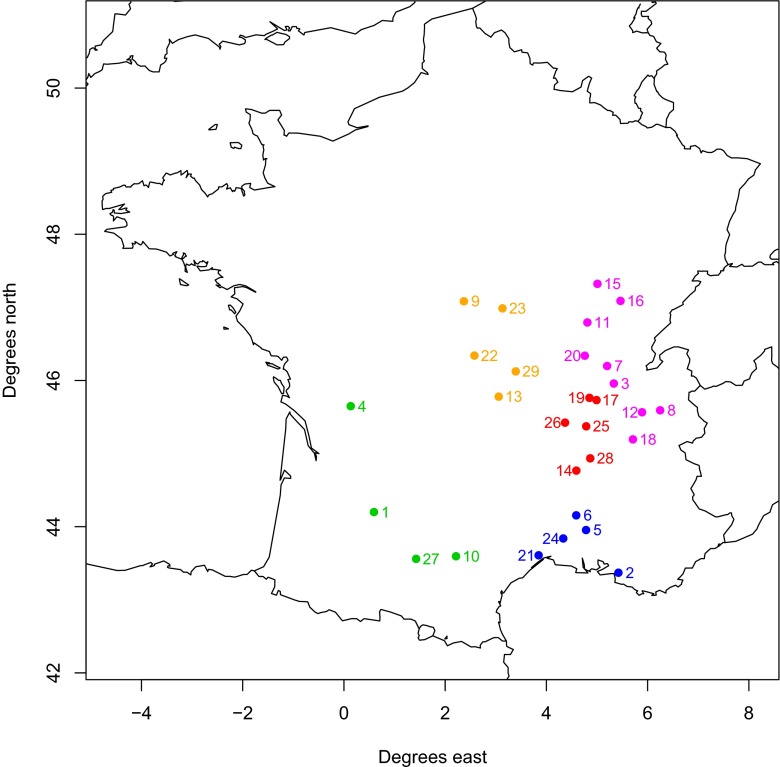
Sites where pollen concentrations are recorded. The numbers refer to the numbering in Table S.1. The coloring denotes the regions that are introduced during the analysis of the results: sites in region A are colored in red, sites in region B are colored in blue, sites in region C are colored in green, sites in region D are colored in orange, sites in region E are colored in pink

The simulated ragweed pollen concentrations were compared to measured values at French pollen observational sites (Table S.1 in the supplementary materials and Fig. 2). The pollen data were recorded at the RNSA (Réseau National de Surveillance Aérobiologique). The measured pollen concentrations are available in a daily resolution for the entire pollen season of 2012.

### Calibration of the maps

The different approaches to generate the pollen source map lead to different descriptors of pollen amount: the map INV-# contains the number of plants per grid cell, the map INV-yn shows the number of plant locations per grid cell while the map LUPC gives the plant density of the grid cell with respect to the maximum density observed in Europe (which occurs near Kecskemét, HU; see Skjøth et al. (2010), Thibaudon et al. (2014)). Likewise, the potential maps yield a plant density in %. This, however, is a derived value based on the climatic suitability of the grid cell regarding ragweed growth. As this was obtained in three different ways, the resulting densities are not directly related to each other. Using the same configuration of the emission parameterization for all maps, it is clear that the raw numbers of the maps have to be calibrated in order to produce meaningful pollen concentrations in the NWP model. The calibration includes the following steps for each of the maps separately:

1. Simulation of the entire pollen season of 2012 using the non-calibrated maps.
2. Comparison of the simulated and measured mean daily pollen concentrations: The overall level of the distribution map should reproduce the pollen level under ideal conditions (e.g., during the height of the pollen season, optimal emission conditions, ...). Assuming that such ideal conditions should lead to relatively high pollen concentrations, only days with at least 40 pollen per cubic meter (daily mean) are used for the calibration. Taking this subset of simulated and measured pollen concentrations, the ratio between simulated and observed values is calculated.
3. The non-calibrated maps are then calibrated by multiplication with this ratio.

### Comparison of simulated and observed pollen concentrations

The simulated daily mean pollen concentrations resulting from the different maps were statistically compared to measured concentrations in France. For each of the maps and each of the observational sites, we calculated the following categorical scores based on 2 ×2 contingency tables using two different thresholds (5 and 20 pollen per cubic meter of air, representing low and strong pollen concentrations, respectively) to define a pollen event: Pierce Skill Score (PSS), Threat Score (TS), False Alarm Ratio (FAR). Additionally, we computed the correlation coefficient r and its corresponding *p*-value, the root-mean-square-error (rmse), the fractional bias (FB), and the index of agreement *d*_1_. Table 1 gives an overview about these scores. For a more detailed description, please refer to Zink et al. (2013), Zink (2014), Wilks (2006), and GAW Report No 181 (2008).

**Table 1:** Overview about the statistical scores used in this study

Score	Range	Interpretation
PSS	−1 to 1	best: 1
		random: 0
		worst: -1
TS	0 to 1	best: 1
		worst: 0
FAR	0 to 1	best: 0
		worst: 1
r	−1 to 1	perfect correlation: 1
		no correlation: 0
		perfect anti-correlation: -1
p-value	0 to 1	significant result: <0.05
*d*_1_	0 to 1	best: 1
		worst: 0
FB	not restricted	best: 0
rmse	not restricted	best: 0

## Results

In a first step, we compare the resulting maps visually as this is a good method to get an overview of the results. In our opinion, the human eye is still one of the best devices to compare different images. We are well aware that this approach is somewhat subjective. Therefore, we provide an objective statistical analysis as a second step.

### Visual comparison of the maps

**Fig. 3:**
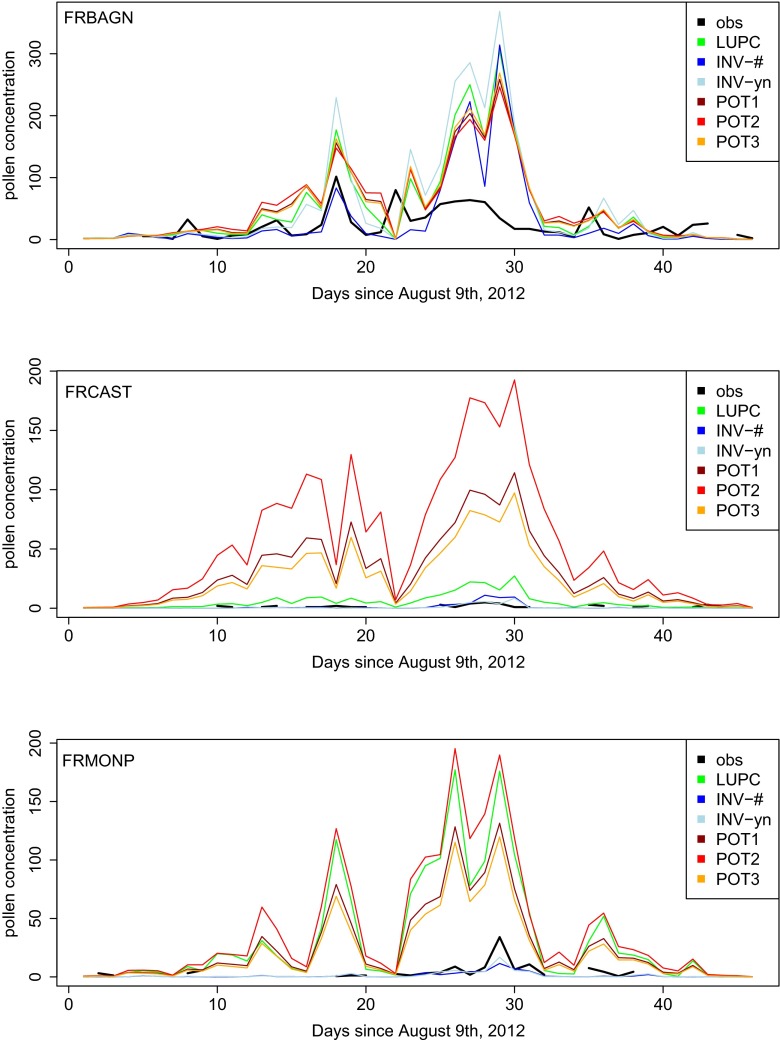
Time series of observed and simulated pollen concentrations at exemplary observational sites based on simulations using the calibrated maps. The concentrations are given as daily mean values in pollen per cubic meter of air

We have first conducted a visual comparison of the non-calibrated maps. They were plotted using a logarithmic scale (Fig. 1). This was done since ragweed shows very inhomogeneous plant densities across France: in some areas (e.g., the western part of Rhône-Alpes, red sites in Fig. 2), the densities/numbers of plants are very high, while in other areas (e.g., northwestern France), the densities/numbers of plants are very low. Map POT2 only gives presence/absence information and thus the quantitative information of this map cannot be compared to the other five maps. Since the spatial extent of the ragweed distribution in POT2 equals that in POT3, we will refrain from describing map POT2 at all. The visual comparison of the remaining five maps yields the following findings:

- The western part of the region of Rhône-Alpes is one of the major ragweed pollen sources in Europe (Thibaudon et al. 2014) which is reproduced in all of the maps even if the details differ largely. In map LUPC, the area of maximum plant density is rather small, while it is more important and reaching further south in maps INV-# and INV-yn. In the potential maps POT1 and POT3, this western part of Rhône-Alpes is also very strongly populated with ragweed. However, the area is a lot larger, stretching from the Mediterranean coast to the borders of Germany. The gradients within the highly populated area are not as strong as in map LUPC.
- The Mediterranean coast is nearly free of ragweed plants in the maps INV-# and INV-yn, while in map LUPC, it displays a continuous ragweed density of up to 10 % and in map POT1 it is even more strongly populated. Map POT3 also displays a dense ragweed growth between Marseille and Montpellier, but the high densities of up to 30 % do not totally reach the coastline. This visual impression is reflected in the time series of simulated and observed pollen concentrations for 2012 (compare Fig. 3): In Montpellier (FRMONP), maps LUPC, POT1, POT2, and POT3 strongly overestimate the pollen concentrations while maps INV-# and INV-yn underestimate them. Further north (e.g., site FRBAGN), all six maps overestimate the observed pollen concentrations.
- A region in central France (close to the cities Bourges and Nevers, sites 9 and 23 in Fig. 2) is moderately populated in maps INV-#, INV-yn, and LUPC but their the exact positions do not match. In the maps POT1 and POT3, this region belongs to the highly infested areas with densities of up to 30 %.
- In maps INV-# and INV-yn, the river Loire can easily be identified as a line source of ragweed pollen. A small part of the river (from Nevers, site 23, northwestwards) is also captured as a ragweed pollen source in maps LUPC, POT1 and POT3.
- While in the maps INV-# and INV-yn, the greater part of France is free of ragweed plants, these regions display a low plant density in map LUPC. The densities only rarely exceed 3 % and are below 1 % for most of the area. Map POT1 does not display areas free of ragweed at all. Map POT3 shows larger areas with no or only small ragweed populations. The gradient between these ragweed free areas to the neighboring populated areas is rather strong going from 0 to more than 10 % within only a few grid cells. The time series of pollen concentrations at site 10 (FRCAST, Fig. 3) reflects this impression: while the inventory maps display rather low pollen concentrations, map LUPC shows moderate overestimation and the potential maps show very strong overestimations.
- A comparison between the maps INV-# and INV-yn is rather corollary: obviously, the spatial ranges of ragweed populations coincide since the maps are based on the same plant locations. Using only presence/absence information (map INV-yn) assimilates the gradients within this spatial range.

Overall, the coarse distribution (big populations in western Rhône-Alpes and none or only very small populations in northern and northwestern France) is reflected in all of the maps. However, when looking at smaller scales, both the spatial patterns and the plant densities in certain areas vary considerably between the different maps.

### Statistical analysis of simulated pollen concentrations

The statistical analysis of the simulated pollen concentrations was done for each observational site separately. The result is rather divers: it depends both on the score and on the site whether a specific map performs well or not. In order to enhance the representativeness of the results, we have grouped the observational sites into five regions. This was done because simulated pollen concentrations represent the mean areal values while observations are point values.

- **Region A** represents the main pollen source region in France including the observational sites 14, 17, 19, 25, 26, and 28 (red dots in Fig. 2).
- **Region B** is the area south of the main source stretching to the Mediterranean coast (observational sites 2, 5, 6, 21, and 24; blue dots in Fig. 2), which displays different levels of ragweed infestation depending on the map.
- In **Region C** are the observational sites 1, 4, 10, and 27, located to the west and southwest of the main source and fairly free of ragweed plants (green dots in Fig. 2).
- **Region D** contains the observational sites 9, 13, 22, 23, and 29 that are located to the northwest of the main source and display an intermediate level of ragweed infestation (orange dots in Fig. 2).
- **Region E** is located to the north and northeast of the main source and displays inhomogeneous ragweed infestation depending on the map (observational sites 3, 7, 8, 11, 12, 15, 16, 18, 20; pink dots in Fig. 2).

The statistical results of the individual sites were averaged to obtain regional values. The exact values for each statistical score and region (Tables S.2 to S.6) and the mean values over all sites (Table S.7) can be found in the supplementary materials. There, we also provide some considerations concerning the interpretation and some detailed observations when comparing the individual scores.

**Fig. 4:**
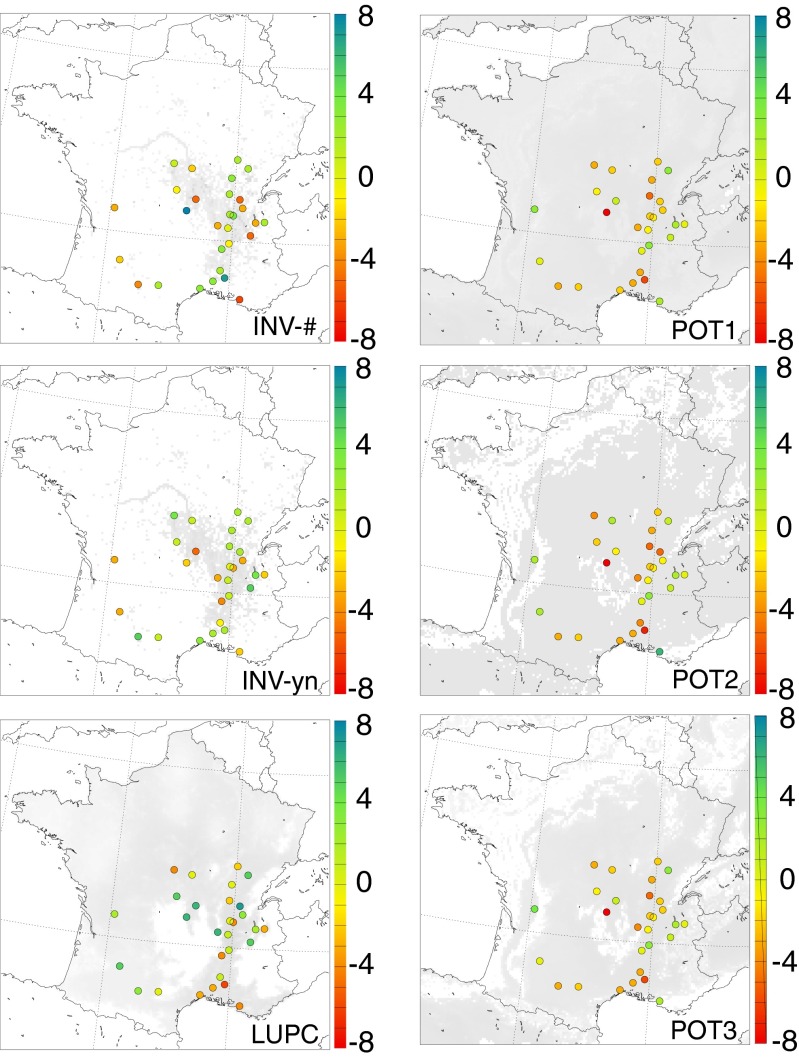
Sums of points representing the goodness of each map (see explanations in Chapter “Statistical analysis of simulated pollen concentrations”). Good results are displayed in green colors, bad results in red colors. The scale gives the number of points that each map scores at each observational site. The background shading in grey represents the distribution map

In order to achieve our original goal, i.e., to judge the quality of the distribution maps based on the statistical scores from the comparison to observed values, some objective method to combine the information from all the scores is required. However, it seems difficult to deduce a unique ranking of the maps using ’some averaging’ of the raw values of the statistical scores. Often, the results of several maps are rather close, thus taking the best score only would neglect methods nearly as good as the best. This would randomly favor one method above another. We have therefore devised a ’grading system’ based on attributing points to good/medium/bad statistical performance. For this, we have determined the actual range of values for each score and site/region. This range is divided into three equally sized portions (terciles) that are used to assess the distribution maps: if the result of a specific map falls into the best third of the range, the map scores one point. If it falls into the worst third of the range, one point is taken from the map. If it falls into the middle third, points are neither rewarded nor taken. Sometimes all six maps have very similar scores (e.g., 0.52, 0.53, and 0.54 for a score that can take values between 0 and 1). In such a case, a division of the actual range of values would result in very small portions for each tercile and thus a rather coincidental assignment of points. To avoid this, a score was not taken into account if the actual range of values stretches over less than 5 % of the maximum possible range of values. For example, for the score TS, that can take values between 0 and 1, points were only rewarded (or taken) if the difference between the actual maximum and minimum values is more than 0.05. The points have been assigned twice: (1) for the statistical results of each individual observational site, and (2) for the regional mean values of the statistical scores. This provides a somewhat objective ranking of the distribution maps for the individual sites and for the five regions: the more points the better. Figure 4 shows the number of points at each site for the six distribution maps. The regional ranking can be found in the first five rows of Table 2. Finally, two overall rankings are derived by summing up the points for (1) the 29 observational sites and (2) the five regions for each map separately. The result is displayed in the two last rows of Table 2 (’Total sites’ and ’Total regions’).

**Table 2:** Ranking of the different distribution maps based on the statistical scores calculated for five French regions. For each region, the best map (or maps if several maps have the same rank) is highlighted with the worst map/maps is/are highlighted with . The total ranking of the maps is calculated twice: ’Total regions’ based on mean statistical scores for each region and ’Total sites’ based on the individual results at each observational site

Region	LUPC	INV-#	INV-yn	POT1	POT2	POT3
A	−1	1	−3	−3	−3	−3
B	−4	1	−1	−2	−3	−2
C	3	−2	−1	−1	−1	0
D	4	0	−1	−6	−6	−5
E	0	1	1	−3	−4	−3
Total regions	2	1	−5	−15	−17	−13
Total sites	23	3	9	−39	−42	−40

The ranking of each map for the individual observational sites is displayed in Fig. 4. The first impression of the plots leads to a division of the maps into two groups: maps INV-# and LUPC display all possible results (colors ranging from red to dark green/blue with practically all colors in-between) while the other four maps show a tendency to be somewhat intermediate with colors ranging from red to light green. The good scores in particular (dark green colors) concentrate largely on the maps INV-# and LUPC. Overall, the plots are quite scattered and it is difficult to give clear statements. Only in a few regions, the plots are quite clear: in region B, INV-# scores best except for site FRAIXP which is best in map POT2. Region C and the three southern sites of region D are best in map LUPC. For the remaining sites, the ranking of the different maps varies between the sites.

Looking at the regional ranking (Table 2), map LUPC, which is the overall best scoring map, performs quite ambiguously: in the regions C and D, it has by far the best rank. In region B, it has the worst result of all maps while in the other two regions it is intermediate. The potential maps clearly score worst: they never have the highest number of points and in almost all cases one (or more) of the potential maps has the smallest number of points. Within the potential maps, map POT2 shows the worst results. The two inventory-based maps display rather similar results in the regions C, D, and E with a maximum difference of one point per region. In region E, both maps have the same best result of all maps. In the regions A and B, map INV-# has the best score of all six maps while map INV-yn is as equally bad as the potential maps in region A and intermediate in region B. In region C, map INV-# scores worst while INV-yn is intermediate.

Calculating the overall ranking in two different ways (based on individual results—’Total sites’—and based on regional mean values—’Total regions’, Table 2) reveals an interesting fact: the order of the maps is similar (LUPC being the best map, followed by the two inventory-based maps, and finally the potential maps)—but the details differ largely. Looking at ’Total region’, the maps LUPC and INV-# obtain nearly the same score (map LUPC being slightly better). Judged on the ’Total sites’, map LUPC has by far the best result. Additionally, for ’Total regions’, map INV-yn is clearly inferior to map INV-#. In contrast, when looking at ’Total sites’, map INV-yn is considerably better than map INV-#. This is in accordance to the result of Zink (2014) who have found that precise quantitative information in the inventory does not necessarily add value to the pollen forecast. In both rankings, the three potential maps have very similar poor results with map POT2 being the worst.

## Discussion and conclusions

We have simulated the ragweed pollen season of 2012 for the region of France using six different distribution maps. The goal was to assess the suitability of each of these maps regarding their use in a numerical pollen forecast. Regarding the statistical results for the individual sites and for the five study regions (Tables S.2 to S.7 in the supplementary materials), the first conclusion is that none of the maps is the best for all cases. Which of the maps scores best depends on the region, and even on the site within the same region. However, in nearly all cases, the three potential maps (POT1, POT2, and POT3) exhibit the lowest scores. This might have been expected given the fact that they represent model predictions (ecological modeling) whereas INV-#, INV-yn, and LUPC reflect more directly the observed densities. Except for some of the sites in regions with big ragweed populations, the potential maps largely overestimate the plant density. This is, of course, due to the methodology that renders the climatic potential for ragweed growth following unlimited dispersal and no-eradication. Plant density is not restricted by land use or soil properties.

Assigning points according to the scores of the specific map relative to the scores of the other maps leads to a clear ranking: LUPC is the best map, followed by the two inventory maps, followed by the three potential maps. However, only in one of the two versions to compute the ranking, the LUPC map is clearly on the lead. Using the regional mean values, map INV-# is almost as good as LUPC. In particular, it is noted that the overall best map (LUPC) is not top in Region A, i.e., the region with the strongest ragweed sources where accurate pollen forecasts (and hence optimal maps) might be most needed. Region B, where the overall best map performs worst may be judged a region with potentially strong influence of transport due to topography (Rhone Valley) and the relatively small distance to the sea. Thus, the results for Region B might not be very conclusive with respect to our goal.

Map INV-# may be judged ’overall second’ as it shows very good results for many regions. However, this kind of map is rather impractical: It depends on a domain-wide and up-to-date assessment of the plant populations. Additionally, the size of the plant populations needs to be estimated. For annual plants such as ragweed all this is critical since the information would have to be updated annually. Only if INV-# would have scored ’best by far’ the discussion about how to make this information available would have been warranted.

The least successful maps POT1, POT2, and POT3 only take climate information into account to derive the po-tential distribution of ragweed. Apparently, the potential overestimates the actual plant density since not everywhere where the plant could grow it actually does. Nevertheless, one may expect that using an extended potential map (considering both land use and climate information) as a basis and tuning it with SPIs would possibly render a map that is considerably better than any of the maps used in this study. Given the fact that in essence LUPC is a very simple potential map (taking into account land use only) tuned with mean SPIs, this therefore suggests a potential pathway to further improve the LUPC approach.

Overall, the present simulation results suggest that for numerical pollen forecasts—at least for pollen originating from annual plants—an approach based on a combination of land use information and pollen data (SPI) is best. This certainly has the immediate advantage that land use information is by default available for the entire domain of a NWP model. The success of the LUPC map, however, is also critically dependent on the availability of pollen observations. This emphasizes the importance of maintaining and extending these networks—if ever possible in an automated manner.

The main drawback of using SPIs for the tuning of a distribution map is probably the fact that they are influenced not only by locally emitted pollen but also by transported pollen (see results in Region B with LUPC). In order to eliminate transport influences, a careful ’calibration strategy’ (only using days with assumed minimal influence of transport rather than the entire season) might be promising. As a direct pathway in this direction is should be tested whether using a high percentile value (90 % percentile, say) rather than the mean (for which the SPI is an estimator) of the observational information for calibration would be advantageous. In the present example such an approach might also alleviate the relatively bad performance of the LUPC map in the major source region.

Finally, ragweed is an annual plant that is spreading on the one hand and being eradicated on the other hand. Because of this, the distinct distribution and densities can vary largely from year to year. This potentially reduces the performance of all distribution maps based on either land use, climate information and/or SPI. Consequently, numerical pollen forecasts would presumably profit from the use of an assimilation cycle that could respond to annual variations rapidly.

## Electronic supplementary material

Below is the link to the electronic supplementary material.

(PDF 89.3 KB)
