# Supplementary material for: Numerical ragweed pollen forecasts using different source maps: a comparison for France
Source: Int J Biometeorol. 2016 Jun 18;61(1):23–33. doi: 10.1007/s00484-016-1188-x (PMC5179590; doi:10.1007/s00484-016-1188-x)
Supplement: Supplementary file 1 — (PDF 89.3 KB) [file 484_2016_1188_MOESM1_ESM.pdf]

Supplement to
'Numerical ragweed pollen forecasts using different source maps: a comparison for France'

Content:

- List of acronyms.
- A detailed description of the species distribution models used to generate the potential maps.
- Table S.1 gives details about the observational sites used in the study.
- Considerations concerning the interpretation and some detailed observations when comparing the individual statistical scores.
- Table S.2 gives the statistical results for region A.
- Table S.3 gives the statistical results for region B.
- Table S.4 gives the statistical results for region C.
- Table S.5 gives the statistical results for region D.
- Table S.6 gives the statistical results for region E.
- Table S.7 gives the statistical results averaged over all regions.

List of acronyms

COSMO-ART	Consortium for Small-scale Modelling - Aerosols and Reactive Trace Gases
d_1	Index of agreement
FAR	False Alarm Ratio
FB	Fractional Bias
FCBN	Fédération des conservatoires botaniques nationaux
GLM	Generalized linear model
INV-#	Distribution map based on numbered inventory data
INV-yn	Distribution map based on categorical inventory data
LUPC	Distribution map based on land use and pollen count data
Maxent	Maximum entropy method
MESS	Multivariate Environmental Similarity Surfaces analysis
NWP	Numerical Weather Prediction
POT1/POT2/POT3	Distribution maps based on ecological modeling
PSS	Pierce Skill Score
rmse	Root-mean-square-error
RNSA	Réseau National de Surveillance Aérobiologique
SDM	Species distribution model
SPI	Seasonal Pollen Index
TS	Threat Score

Species distribution models - SDMs

Species distribution models (SDMs) were calibrated using occurrences of ragweed from various herbarium and environmental agencies in France and neighboring countries (Switzerland, Germany, Austria, Slovenia and Croatia) where ragweed can accomplish a full reproduction cycle (Storkey et al., 2014). Within the boundaries of these countries, 10.000 points were randomly sampled to depict the climatological conditions of the regions where ragweed is not present. After disaggregation of the data by keeping a minimal distance of 10 km between each pair of occurrences (i.e. to decrease sampling bias and spatial autocorrelation; Verbruggen et al. (2013)), the 1570 occurrences left were attached to 6 climate variables known to be important for the delineation of the potential distribution of ragweed (Gentilini, 2010): maximum temperature of the warmest month, annual temperature range, mean temperature of the coldest quarter, precipitation of the driest quarter, precipitation of the warmest quarter and aridity. Climate data were downloaded at a 30 arc second resolution from the Worldclim database (Hijmans et al., 2005) and aridity data was taken from Trabucco et al. (2008). Different modeling techniques were used: generalized linear models (GLM, with second order polynomial coefficient and stepwise selection using the Bayesian information criteria, McCullagh and Nelder, 1983)), generalized boosted regression models (GBM, Friedman et al., 2000) and the maximum enthropy method (Maxent, Phillips et al., 2006). The predictions of these models were averaged following an ensemble approach (Thuiller et al., 2009). Modeling was achieved using the R package 'biomod2' (Thuiller et al., 2009), keeping the default set of parameters. Models were calibrated on 70% of the data and evaluated with the remaining 30% of the data. The modeling procedure was replicated 10 times and the final results consist in an average of the 10 replicates.

The contributions of the different variables were estimated by assessing the impact of variable randomizations on the predictions (Thuiller et al., 2009). The most important variables in the models are (in the order of decreasing contribution): maximum temperature of the warmest month (drop contribution = 0.56, for explanations see Thuiller et al. (2009)), annual temperature range (0.20), mean temperature of the coldest quarter (0.13), aridity (0.08), precipitation of the driest quarter (0.07) and precipitation of the warmest quarter (0.07).

The SDMs were evaluated with the Area Under the Curve of a Receiver Operating Characteristics (AUC, ROC, Zweig and Campbell, 1993) and the True Skill Statistic (TSS, Allouche et al., 2006). AUC varies between 0 (meaning: complete counter predictions of the model) and 1 (perfect fit with the observed distribution), 0.5 being random predictions. TSS varies as a correlation coefficient between -1 and 1, with -1 meaning counter-prediction, 0 random prediction, and 1 perfect prediction. With a mean AUC of 0.803 +/- 0.009 and a mean value of TSS value of 0.481 +/- 0.017, the predictions of the models can be considered as 'good' (Swets, 1988).

Table S.1: Observational sites for pollen measurements in France. Longitude (Lon) and latitude (Lat) of the each observational site are given.

#	Station	Town	Lon	Lat
1	FRAGEN	Agen	0.6200	44.2000
2	FRAIXP	Aix-en-Provence	5.4500	43.3700
3	FRAMBE	Ambérieu-en-Bugey	5.3589	45.9578
4	FRANGO	Angoulême	0.1611	45.6483
5	FRAVIG	Avignon	4.8102	43.9536
6	FRBAGN	Bagnols-sur-Cèze	4.6164	44.1549
7	FRBOUB	Bourg-en-Bresse	5.2269	46.1977
8	FRBOUJ	Bourgoin	6.2744	45.5917
9	FRBOUR	Bourges	2.3965	47.0829
10	FRCAST	Castres	2.2419	43.5950
11	FRCHAL	Chalon-sur-Saône	4.8369	46.7933
12	FRCHAM	Chambéry	5.9169	45.5652
13	FRCLER	Clermont-Ferrand	3.0850	45.7783
14	FRCOUX	Coux	4.6181	44.7667
15	FRDIJO	Dijon	5.0350	47.3216
16	FRDOLE	Dole	5.4908	47.0881
17	FRGENA	Genas	4.9966	45.7330
18	FRGREN	Grenoble	5.7363	45.1941
19	FRLYON	Lyon	4.8566	45.7633
20	FRMACO	Mâcon	4.7858	46.3383
21	FRMONP	Montpellier	3.8733	43.6083
22	FRMONT	Montluçon	2.6050	46.3400
23	FRNEVE	Nevers	3.1602	46.9861
24	FRNIME	Nîmes	4.3600	43.8383
25	FRROUS	Roussillon	4.8136	45.3727
26	FRSTET	Saint-Étienne	4.3950	45.4227
27	FRTOUS	Toulouse	1.4530	43.5594
28	FRVALE	Valence	4.8931	44.9347
29	FRVICH	Vichy	3.4219	46.1238

Considerations concerning the interpretation and some detailed observations when comparing the individual statistical scores.

The Threat Score (TS) and the False Alarm Ratio (FAR) always have to be seen together: only if both scores show good results, the simulation can really be regarded as good. This is because a simulation that always exceeds the threshold concentration will lead to a perfect TS, even though the usefulness of the forecast is strongly limited if it is not able to forecast low pollen concentrations. This feature can be found in all of the maps: in many cases, the TS values are quite good, largely exceeding 0.5 (e.g., Tables S.2 and S.5). However, FAR values that even reach the worst possible value of 1.0 (Table S.4) show that all simulations strongly overestimate the observed pollen concentrations. This is also reflected in the results for the Pierce Skill Score (PSS) that are rather poor with a maximum of about 0.5 and values down to about 0.1. For most of the maps, the PSS using a threshold of 20 pollen per cubic meter of air is clearly better than the PSS using a threshold of 5 pollen per cubic meter of air. This shows that it is especially difficult to forecast low pollen concentrations. The potential maps (POT1, POT2, POT3) strongly overestimate the pollen concentrations: the fractional bias (FB) reaches values of up to 1.78 for these maps (Table S.4). This is also true for the LUPC map except for region D (Table S.5) where the level of the simulated pollen concentrations is rather close to the observations. Map INV-yn shows varying results: for some regions, the pollen concentrations are estimated relatively well while in other regions they are over- or underestimated. Map INV-# clearly displays the best results for FB. For most regions it underestimates the pollen concentrations. However, the underestimation is rather small and in some regions negligible. The correlation between measured and simulated pollen concentrations differs strongly between the regions: for example, in region C (Table S.4) the correlations are very poor while in region B (Table S.3) they are rather good. However, the p-values of the correlation coefficients denote that only the results for the regions A and B are significant (Tables S.2 and S.3). Within the regions, the differences regarding the correlations are rather small between the six maps. We conclude that the temporal evolution of the pollen concentrations depends rather on the pollen emission (which is parameterized equally for all maps) than on the source map.

Table S.2: Statistical results of the simulations using different ragweed distribution maps for region A. Region A is the main pollen source region in France including the observational sites 14, 17, 19, 25, 26 and 28.

Score	Threshold	LUPC	INV-#	INV-yn	POT1	POT2	POT3
PSS	5	0.13	0.32	0.19	0.22	0.23	0.22
TS	5	0.74	0.68	0.72	0.71	0.72	0.71
FAR	5	0.19	0.17	0.20	0.20	0.20	0.20
PSS	20	0.52	0.47	0.52	0.49	0.49	0.49
TS	20	0.56	0.48	0.51	0.46	0.46	0.46
FAR	20	0.36	0.31	0.37	0.37	0.37	0.37
rmse		144.32	59.46	115.39	60.30	59.85	61.12
FB		0.74	0.08	0.64	0.38	0.34	0.40
r		0.48	0.42	0.42	0.44	0.43	0.44
p-value		0.01	0.08	0.02	0.04	0.04	0.04
d_1		0.38	0.48	0.38	0.41	0.41	0.40

Table S.3: Statistical results of the simulations using different ragweed distribution maps for region B. Region B is the area between the main source (region A) and the Mediterranean coast (observational sites 2, 5, 6, 21 and 24) which displays different levels of ragweed infestation depending on the map.

Score	Threshold	LUPC	INV-#	INV-yn	POT1	POT2	POT3
PSS	5	0.18	0.38	0.22	0.21	0.21	0.15
TS	5	0.55	0.49	0.47	0.55	0.56	0.51
FAR	5	0.41	0.19	0.22	0.39	0.39	0.41
PSS	20	0.33	0.26	0.24	0.34	0.32	0.38
TS	20	0.24	0.22	0.23	0.26	0.26	0.28
FAR	20	0.72	0.50	0.44	0.69	0.68	0.64
rmse		74.33	26.99	32.04	55.65	64.65	53.87
FB		1.21	-0.21	-0.09	1.01	1.04	0.84
r		0.56	0.56	0.65	0.56	0.56	0.58
p-value		0.00	0.01	0.01	0.00	0.00	0.00
d_1		0.19	0.47	0.44	0.25	0.24	0.27

Table S.4: Statistical results of the simulations using different ragweed distribution maps for region C. Region C are the observational sites 1, 4, 10, and 27, located to the west and southwest of the main source and fairly free of ragweed plants.

Score	Threshold	LUPC	INV-#	INV-yn	POT1	POT2	POT3
PSS	5	0.42	0.12	0.25	0.12	0.10	0.14
TS	5	0.18	0.02	0.06	0.14	0.15	0.15
FAR	5	0.78	0.98	0.94	0.85	0.85	0.84
PSS	20	NA	NA	NA	NA	NA	NA
TS	20	0.00	0.00	NA	0.00	0.00	0.00
FAR	20	1.00	1.00	NA	1.00	1.00	1.00
rmse		9.73	30.29	3.60	41.00	81.99	30.97
FB		0.68	-0.36	-1.07	1.57	1.78	1.40
r		0.16	0.10	0.10	0.18	0.18	0.17
p-value		0.53	0.51	0.43	0.40	0.41	0.42
d_1		0.23	0.29	0.37	0.06	0.03	0.10

Table S.5: Statistical results of the simulations using different ragweed distribution maps. Region D are the observational sites 9, 13, 22, 23 and 29 that are located to the northwest of the main source and display an intermediate level of ragweed infestation.

Score	Threshold	LUPC	INV-#	INV-yn	POT1	POT2	POT3
PSS	5	0.33	0.38	0.33	0.28	0.25	0.28
TS	5	0.53	0.52	0.52	0.54	0.54	0.54
FAR	5	0.29	0.21	0.27	0.33	0.34	0.33
PSS	20	0.43	0.29	0.31	0.33	0.33	0.34
TS	20	0.36	0.25	0.26	0.25	0.28	0.26
FAR	20	0.54	0.62	0.51	0.67	0.66	0.67
rmse		22.60	39.87	46.94	38.28	49.02	36.09
FB		-0.05	-0.19	0.22	0.60	0.82	0.52
r		0.39	0.35	0.44	0.39	0.39	0.40
p-value		0.33	0.22	0.15	0.31	0.34	0.27
d_1		0.50	0.45	0.39	0.33	0.29	0.35

Table S.6: Statistical results of the simulations using different ragweed distribution maps for region E. Region E is located to the north and northeast of the main source and displays inhomogeneous ragweed infestation depending on the map (observational sites 3, 7, 8, 11, 12, 15, 16, 18, 20).

Score	Threshold	LUPC	INV-#	INV-yn	POT1	POT2	POT3
PSS	5	0.16	0.25	0.21	0.17	0.17	0.19
TS	5	0.50	0.45	0.45	0.51	0.52	0.52
FAR	5	0.35	0.29	0.30	0.35	0.35	0.34
PSS	20	0.43	0.19	0.24	0.34	0.31	0.34
TS	20	0.27	0.21	0.22	0.24	0.23	0.24
FAR	20	0.66	0.53	0.45	0.72	0.73	0.72
rmse		30.94	37.93	29.84	39.52	42.90	39.45
FB		0.41	-0.03	-0.18	0.75	0.83	0.74
r		0.30	0.37	0.39	0.22	0.21	0.21
p-value		0.28	0.18	0.18	0.47	0.48	0.47
d_1		0.36	0.42	0.43	0.29	0.28	0.29

Table S.7: Statistical results of the simulations using different ragweed distribution maps. The scores obtained for the different regions (compare Tables S.2 to S.6) are averaged to give the mean value for all of France.

Score	Threshold	LUPC	INV-#	INV-yn	POT1	POT2	POT3
PSS	5	0.22	0.30	0.23	0.20	0.19	0.20
TS	5	0.52	0.46	0.47	0.52	0.52	0.51
FAR	5	0.38	0.33	0.35	0.39	0.39	0.39
PSS	20	0.43	0.29	0.32	0.37	0.36	0.38
TS	20	0.30	0.28	0.30	0.26	0.26	0.26
FAR	20	0.63	0.51	0.44	0.67	0.67	0.66
rmse		57.52	39.78	47.25	46.59	56.60	44.67
FB		0.57	-0.11	-0.05	0.80	0.89	0.74
r		0.38	0.37	0.41	0.35	0.34	0.35
p-value		0.22	0.18	0.15	0.26	0.27	0.26
d_1		0.34	0.43	0.41	0.28	0.27	0.29

References

Allouche, O., Tsoar, A., and Kadmon, R.: Assessing the accuracy of species distribution models: prevalence, kappa and the true skill statistic (TSS), Journal of Applied Ecology, 43, 1223–1232, 2006.

Friedman, J. H., Hastie, T. J., and Tibshirani, R.: Additive logistic regression: a statistical view of boosting, Annals of Statistics, 28, 337–374, 2000.

Gentilini, E.: Modelling the distribution and spread of Common Ragweed (Ambrosia artemisiifolia) at multiple scales, Master's thesis, University of Lausanne, 2010.

Hijmans, R. J., Cameron, S. E., Parra, J. L., Jones, P. G., and Jarvis, A.: Very high resolution interpolated climate surfaces for global land areas, International Journal of Climatology, 25, 1965–1978, 2005.

McCullagh, P. and Nelder, J. A.: Generalized Linear Models, Monographs on Statistics and Applied Probability, Springer US, 1 edn., doi:10.1007/978-1-4899-3244-0, 1983.

Phillips, S. J., Anderson, R. P., and Schapire, R. E.: Maximum entropy modeling of species geographic distributions, Ecological Modelling, 190, 231–259, 2006.

Storkey, J., Stratonovitch, P., Chapman, D. S., Vidotto, F., and Semenov, M. A.: A process-based approach to predicting the effect of climate change on the distribution of an invasive allergenic plant in Europe, PloS one, 9, e88 156, 2014.

Swets, J. A.: Measuring the accuracy of diagnostic systems, Science, 240, 1285–1293, 1988.

Thuiller, W., Lafourcade, B., Engler, R., and Araújo, M. B.: BIOMOD – a platform for ensemble forecasting of species distributions, Ecography, 32, 369–373, 2009.

Trabucco, A., Zomer, R. J., Bossio, D. A., van Straaten, O., and Verchot, L. V.: Climate change mitigation through afforestation/reforestation: A global analysis of hydrologic impacts with four case studies, Agriculture, Ecosystems & Environment, 126, 81–97, 2008.

Verbruggen, H., Tyberghein, L., Belton, G. S., Mineur, F., Jueterbock, A., Hoarau, G., Gurgel, C. F. D., and De Clerck, O.: Improving Transferability of Introduced Species' Distribution Models: New Tools to Forecast the Spread of a Highly Invasive Seaweed, PloS one, 8, e68 337, 2013.

Zweig, M. H. and Campbell, G.: Receiver-operating characteristic (ROC) plots: a fundamental evaluation tool in clinical medicine., Clinical Chemistry, 39, 561–577, 1993.
